# Antigen-Antibody Affinity for Dry Eye Biomarkers by Label Free Biosensing. Comparison with the ELISA Technique

**DOI:** 10.3390/s150819819

**Published:** 2015-08-13

**Authors:** Maríafe Laguna, Miguel Holgado, Ana L. Hernandez, Beatriz Santamaría, Alvaro Lavín, Javier Soria, Tatiana Suarez, Carlota Bardina, Mónica Jara, Francisco J. Sanza, Rafael Casquel

**Affiliations:** 1 Center for Biomedical Technology, Optics, Photonics and Biophotonics Lab, Universidad Politécnica de Madrid. Campus Montegancedo, 28223 Pozuelo de Alarcón, Madrid, Spain; E-Mails: m.holgado@upm.es (M.H.); ana.lopez@upm.es (A.L.H.); beatriz.santamaria@ctb.upm.es (B.S.); alavin@etsii.upm.es (A.L.); fj.sanza@upm.es (F.J.S.); rafael.casquel@upm.es (R.C.); 2 Department of Applied Physics and Material, Escuela Técnica Superior de Ingenieros Industriales (ETSII), Universidad Politécnica de Madrid, Jose Gutiérrez Abascal, 2. 28006 Madrid, Spain; 3 BioOpticalDetection, Centro de Empresas de la UPM, Campus Montegancedo, 28223 Pozuelo de Alarcón, Madrid, Spain; 4 Bioftalmik. Parque Tecnológico Zamudio Ed. 800 2ª Planta 48160, Bizkaia, Spain; E-Mails: javier.soria@bioftalmik.com (J.S.); tatiana.suarez@bioftalmik.com (T.S.); 5 AntibodyBcn, MRB 104 Modul b UAB Campus, 08193 Bellaterra, Barcelona, Spain; E-Mails: carlota.bardina@antibodybcn.com (C.B.); monica.jara@antibodybcn.com (M.J.)

**Keywords:** photonic sensing cells BICELLs, biosensors, label-free immunoassay, dry eye biomarkers

## Abstract

The specificity and affinity of antibody-antigen interactions is a fundamental way to achieve reliable biosensing responses. Different proteins involved with dry eye dysfunction: ANXA1, ANXA11, CST4, PRDX5, PLAA and S100A6; were validated as biomarkers. In this work several antibodies were tested for ANXA1, ANXA11 and PRDX5 to select the best candidates for each biomarker. The results were obtained by using Biophotonic Sensing Cells (BICELLs) as an efficient methodology for label-free biosensing and compared with the Enzyme-Linked Immuno Sorbent Assay (ELISA) technique.

## 1. Introduction

As reported by Lemp *et al.* [1], dry eye disease is a multifactorial chronic disorder of the ocular surface that affects up to 100 million people worldwide. Diagnosis and management of dry eye has been a source of frustration to clinicians for a lack of correlation between signs and symptoms. Dry eye (DE) and meibomian gland dysfunction (MGD) are common inflammatory ocular surface diseases affecting tear film stability and ocular surface integrity. The pathophysiology of both conditions is complex and thought to represent the interaction of multiple mechanisms including tear film hyperosmolarity, instability, and subsequent activation of an inflammatory cascade, with release of inflammatory mediators into the tears, which in turn can damage the ocular surface epithelium.

Label-free optical biosensors have been demonstrated to be a good technology for *In-Vitro* Diagnostics (IVD) due to advantages *versus* labeled techniques [2,3]. The short turnaround and cost-effectiveness advantages are very important factors for final users and health professionals as a whole. Mainly, three important factors are connected with the Limit of Detection (LoD) of optical label-free biosensing: the transducer sensitivity, resolution of the optical reader and the performance of the immunoassay. The latter one, the antigen-antibody interaction, plays an important role to achieve a competitive LoD. In this sense, the study of specificity and affinity of antibody-antigen interactions is fundamental for understanding the biological activity of these proteins, as well as to develop suitable biosensors.

As it is well explained [4,5], a highly specific bimolecular association is achieved by the interaction between an antibody with its corresponding antigen, which involves various non-covalent interactions between the antigen epitope and the variable region of the antibody molecule. These interactions (ionic bonds, hydrogen bonds, hydrophobic interactions and van der Walls interactions) are needed for a strong antigen-antibody binding requiring a high degree of complementarity between antigen (Ag) and antibody (Ab).

Affinity is the strength of binding of a single molecule to its corresponding ligand. Typically it is determined by the equilibrium dissociation constant (K_D_), which is used to evaluate biomolecular interactions. The measurement of the reaction rate constants can be used to define an equilibrium or affinity constant (1/K_D_).Thus, the smaller the K_D_ value, the greater the affinity of an antibody with its target. Antibodies with high affinity have an association constant K_a_ > 10^7^ M^−1^ [6,7].

Biomarkers are frequently used in clinical trials of therapeutics for the assessment of disease states and also for evaluating diagnostic devices. In previous works, several biomarkers where validated for dry eye disease: S100A6, CST4, MMP9, PRDX5, ANXA1, ANXA11, PLAA [8].

In previous articles, our research group has also proven an efficient methodology for label-free biosensing by using Biophotonic Sensing Cells (BICELLs) [9,10], and particularly for dry eye diseases [11]. According to this, in this article we study the affinity of several antibodies for biomarkers: ANXA1, ANXA11, PRDX5 and S100A6 using BICELLs based on SU8 resist Fabry-Perot interferometers with an optical read-out of the biosensor based on the interferometry. 

The label-free optical technique based on BICELLs is a well-reported optical technique where basically changes in the refractive index are produced by the recognition or accumulation events of biomolecules onto the sensing surface [9]. This BICELLs method is a label-free, which means that it is not necessary label-molecules for the detection. However, in the classical Enzyme-Linked Immuno Sorbent Assay (ELISA) protocols a labeled-molecule for subsequent detection is needed.

## 2. Experimental Section

### 2.1. Production of Mouse mAbs

The mAbs were obtained from female Balb/c mice immunized by intraperitoneal injections with the recombinant proteins ANXA1, ANXA11 and PRDX5, separately. The fusion was performed using a Clona Cell-HY kit following the manufacturer’s instructions (Stemcell Technologies, Vancouvert, BC, Canada). Briefly, micesplenocytes were fused with immortal NSO-1 cells (kindly donated by Margaret Goodall, University of Birmingham, Birmingham, UK) with the addition of polyethylene glycol (Clona Cell-HY kit). The resulting mix was grown in selective agar (ClonaCell-HY kit) on 96-well plates. 

Screening of positive hybridoma cell culture supernatant was tested by indirect ELISA. Desired clones were expanded, cultured on a large scale and cryopreserved. The three best hybridomas of each fusion were selected (Table 1) based on its productivity, ELISA signal and growth rate for further studies.

mAbisotypes were determined with the mouse mAbisotyping kit (Sigma-Aldrich, Madrid, Spain), and were purified by Protein G (GE Healthcare, Buckinghamshire, UK) affinity column chromatography. Their purity was confirmed by SDS/PAGE. All mAbs were produced and purified by AntibodyBcn (Barcelona, Spain).

**Table 1:** Antibodies selected from each fusion.

Protein	Antibody Selected
ANXA 1	P4D1
	P6D7
	P10B12
ANXA11	P1B11
	P3F9
	P4D9
PRDX5	P3G1
	P5H6
	P9F4

### 2.2. Affinity ELISA Assay

In order to establish which mAb shown a greater affinity to its own antigen, calibrating curves were carried out by indirect ELISA assays as follows. Ninety-six-well ELISA plates (Santa Cruz Biotech, Dallas, TX, USA) were coated for 4 h at 37 °C with 100 µL per well of each protein in serial dilutions (1:2) from 200 ng/mL to 3.125 ng/mL in 0.2 M carbonate buffer (pH 9.6). Washing was done using 0.05% Phosphate Buffer Saline (PBS)-Tween 20 (PBS-T). Wells were blocked with 2.5% non-fat milk-PBST overnight at 4 °C. Afterwards plates are incubated with 100 µL purified mAbs at 5 μg/mL for 1 h at 37 °C. Ab binding was detected with HRP-conjugated anti-mouse IgG (HRP stands for Horseradish Peroxidase; 1:500 in PBS-T; Santa Cruz Biotech), followed by color development with tetramethylbenzidine ELISA substrate (TMB; Thermo Fisher Scientific, Uppsala, Sweden). The reaction was stopped with 1 M HCl and read at 450 nm by a Multiscan FC microplate reader (Thermo Fisher Scientific).

### 2.3. Biosensor

For this experimental work we used, as photonic transducer, a Biophotonic Sensing Cell (BICELL) based on Fabry-Perot interferometers of SU8 polymeric resist that exhibits a sensitive optical label-free biosensing capability. The Fabry-Perot interferometer is the biotransducer of the biosensor itself. Bicells are based on different type of interferometers and are normally square sensing areas where the recognition events take place. For this particular case, the interferometer employed is a single SU8 layer Fabry-Perot interferometer where part of the light is transmitted through the SU8 reaching the substrate. As a result the interference is produced by the mixed beams coming from the SU8 (and its biomolecules) and the substrate. The large number of interfering beams produces an interferometry profile with a high resolution suitable for biosensing.

We employed SU8 2000.5 (Microchem Corp., Newton, MA, USA) diluted in cyclopentanone [12] for the fabrication of BICELLs. The SU8 resist was deposited by spinning at 3000 rpm for 3 min, then the film was soft-baked at 70 °C for 1 min. An exposure to UV light process was then carried out, followed by a post-bake step at 70 °C for 5 min in order to give a stable thin film. The SU8 surface of the BICELLs was treated with sulfuric acid (95% for 10 s) in order to have a hydrophilic sensing surface. As a result of this treatment, the SU8 epoxy groups are opened and suitable to immobilize covalently the protein [13].

By monitoring the changes in the interferometric profile of theoptical mode response, the immobilization of protein and the recognition of several antibodiescan be properly monitored. Therefore, it is possible to detect the response of the antibody for each biomarker.

### 2.4. Optical Characterization of the Biosensor and Sensing Principle

**Figure 1:**
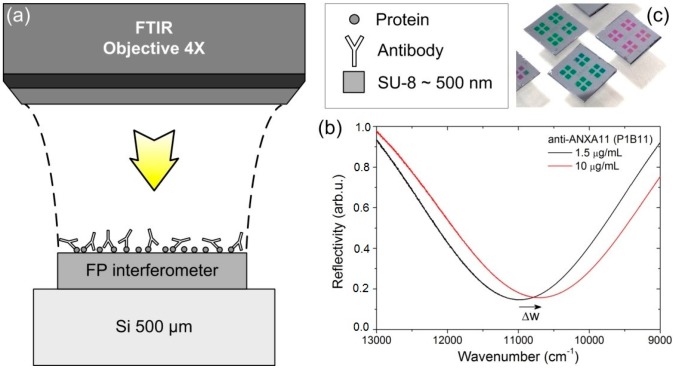
(**a**) Optical setup for measurements and biochemical diagram of the immunoassay; (**b**) optical response for the BICELLs; (**c**) Bicells used in the immunoassay.

The optical readout of the biosensor was accomplished by a Fourier transform visible-infrared (FT-VIS-IR) spectrometer (Vertex 70 adapted to the visible range, Bruker, Madrid, Spain) after each incubation/washing step. We followed the well-described procedure very recently reported in the literature [9] (see in Figure 1a–c).

### 2.5. Immunoassay Procedure

The indirect immunoassay Protein (ANXA1, ANXA11 and PRDX5)/antibody was carried out by a covalent binding of the protein onto the BICELLs SU8 sensing surface until saturation for testing the best clone obtained for AntibodyBcn (Barcelona, Spain). The covalent bond occurs between epoxy ring of SU8 and amine groups of proposed proteins. The incubation of proteins was made until saturation with a volume of 60 µL, with a concentration of 50 µg·mL^−1^ in phosphate buffered saline (pH 7.4,), and at temperature of 37 °C during 20 min. Then, the surfaces were rinsed with deionize water (DI-H_2_O) and blown with dry and clean dust-less air under clean environment.

Avoid nonspecific adsorption is a very important step. In fact, the blocking step avoids the unspecific bounding, especially important for direct immunoassay, where the antibody is firstly immobilized onto the sensing surface. However, for this article, we did not consider using a blocking step because we immobilized the biomarker(indirect immunoassay) until saturation, supposing that the sensing surface is completely filled with the protein (there are a biofilm of protein according with our previous simulations).

Then, we proceeded to recognize the corresponding antibody. The recognition curve of antibody with concentrations 0.2, 0.5, 1.5, 2.5, 5, 10, 25, 5, 10, 25, 50 and 100 µg·mL^−1^ in PBS-pH 7.4 was observed at 37 °C for 20 min for each incubation step. Thus, for each antibody concentration the corresponding BICELLs were washed with PBS-T and water and blown with dry and clean dust-less air.

## 3. Results and Discussion

### 3.1. Results Obtained by ELISA Technique: Affinity Analysis by ELISA

**Figure 2:**
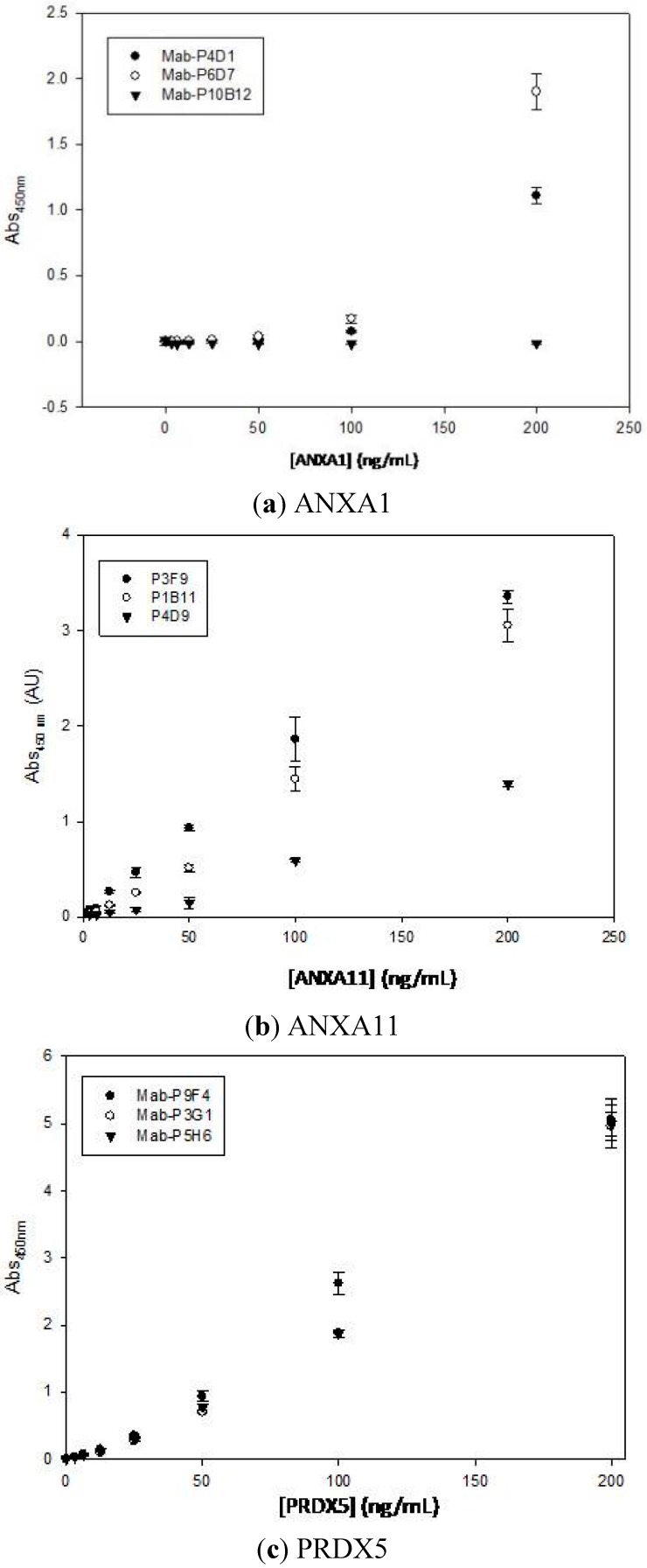
Calibration curves for selected monoclonal antibodies. The absorbance measurements are plotted against the protein concentration ranging from 3.125 ng·mL^−1^ to 200 ng·mL^−1^: (**a**) ANXA1; (**b**) ANXA11; (**c**) PRDX5.

Selected monoclonal antibodies were individually characterized to determine which of them showed the highest affinities which meant strong binding ability to their antigen and would lead to its strong applied value in areas such as detection and diagnosis. Thus, for ANXA 1 (Figure 2a), the antibody P10B12 did not show a significant signal even at high ligand concentrations. The other two antibodies shown a slightly improvement, being antibody P6D7 a little better than the antibody P4D1, with dissociation constants K_D_ of 2.40 μM, and 27.01 μM, respectively. Both antibodies give signals too far from the saturation range, however both antibodies could be used for ANXA1 detection.

In the case of monoclonal antibodies against protein ANXA11 (Figure 2b) all of them showed apparently good signals; both P3F9 and P1B11 are close to the saturating point at the highest ligand concentration employed in the assay. Although P3F9 demonstrated the best ability to bind to antigen ANXA11, P1B11 and P4D9 could be also used for an effective detection of the protein. The dissociation constant (K_D_) of P3F9, P4D9 and P1B11 were 19 nM, 4.87 µM and 1.56 µM, respectively.

Finally in the case of antibodies the intensity shown by the three selected antibodies against PRDX5 (Figure 2c) reveals a high affinity of all of them. Antibody P9F4 has the higher affinity to PRDX5 with a K_D_ of 17.66 nM. Both P3G1 and P5H6 antibodies have a similar affinity rate with a K_D_ of 22.05 nM and 27.01 nM, respectively.

### 3.2. Results Obtained by Optical Label-Free Technique

**Figure 3:**
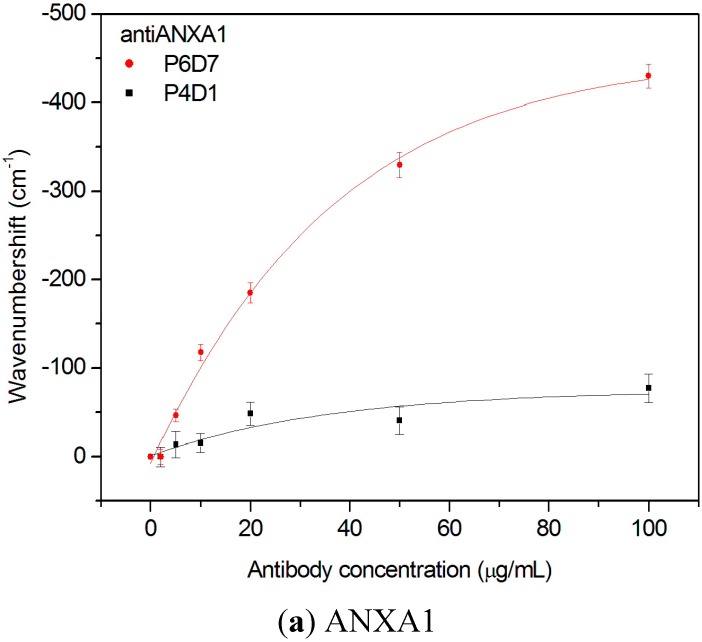

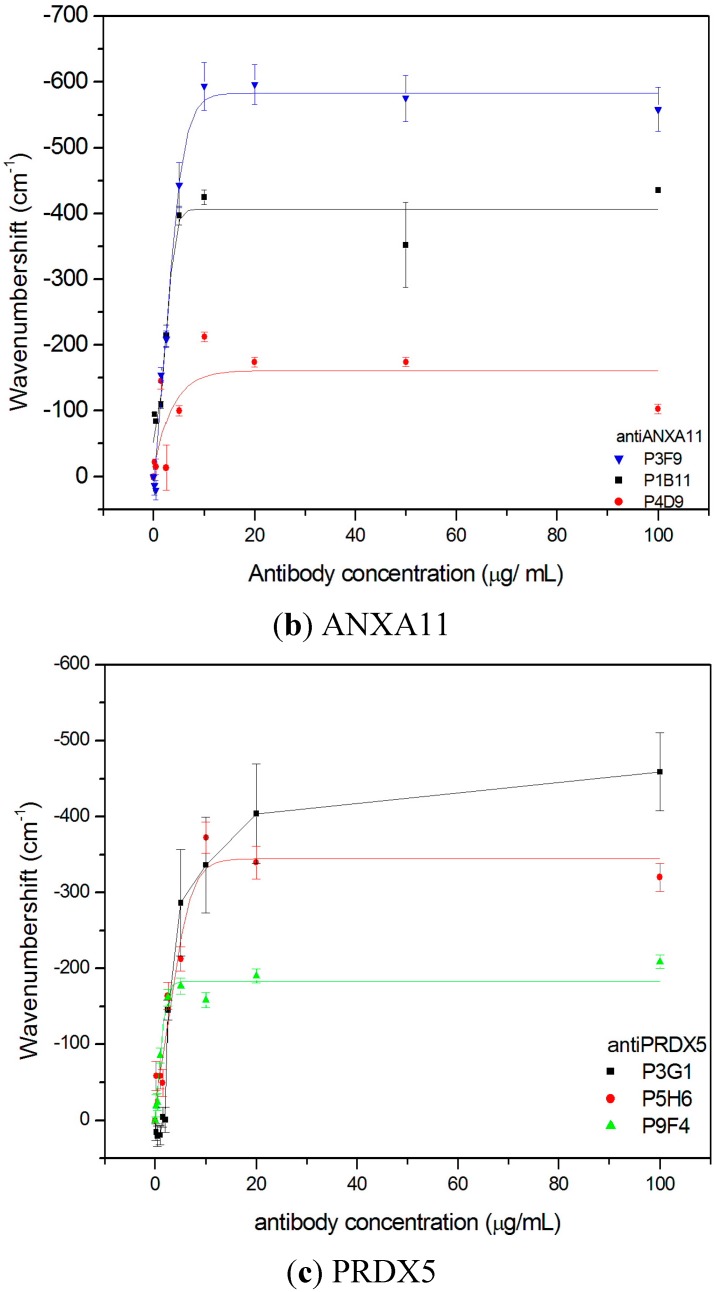
Dip shift against antibody concentration for (**a**) ANXA1; (**b**) ANXA11; (**c**) PRDX5.

In order to analyze the response of the antibody for each biomarker, we evaluated the spectral response for different concentrations of antibody. Figure 3 shows the measured interference dip wavenumber displacement of Fabry-Perot interferometer for increasing concentrations of the different antibodies. In the analyte-receptor recognition reaction, the dissociation constant is expressed as K_D_ = [A]·[R]/[AR], where [A] is the free analyte concentration, [R] is the free receptor concentration and [AR] is the analyte-receptor complex concentration. At the equilibrium, K_D_ = k_d_/k_a_, k_d_ and k_a_ are the kinetic constants for the dissociation and association process, respectively. Thus, K_D_ can be considered as the reciprocal of the analyte affinity towards the receptor. In our experiment the receptor concentration is assumed to be [R] = [R]_total_ − [AR] and when 50% of the binding sites are occupied ([AR] = 0.5·[R]_total_), the dissociation constant is the free analyte concentration K_D_ = [A]. Therefore, the K_D_ value is the antibody concentration causing a response in the transduction equal to 50% of the total transduction change after saturation. In Figure 3a (for ANXA1) two clones were studied (P4D1 and P6D7). The signal for P4D1 clone is much lower than P6D7 clone. Both clones gave an affinity constant values very low (P6D7-K_D_ = 1.6 × 10^−4^ M and P4D1 = 8.86 × 10^−5^ M), resulting in a poor affinity for the protein ANXA1 because antibodies with high affinity must have K_D_ < 10^−7^ M. For these reasons, both antibodies are not considered very good for recognizing the ANXA1 biomarker.

For Anxa11 (Figure 3b) he three antibodies offered a good dynamic range with dissociation constant values lower than 10^−7^ M. The values obtained for P3F9, P4D9 and P1B11 are 20 nM, 15 nM and 33.3 nM, respectively. Figure 3b shows that all antibodies reach the point of saturation below 10 µg·mL^−1^ and the dissociation constants values obtained show the high affinity of the antibodies to its corresponding antigen.

Finally for PRDX5 (Figure 3c), three selected antibodies were studied, showing a high affinity towards PRDX5. The dissociation constants values obtained for P9F4, P5H6 and P3G1 are 7.3 nM, 23.3 nM and 26.6 nM, respectively. These values are in agreement with values obtained by the ELISA technique.

### 3.3. Comparison of the ELISA Technique versus the Optical Label-Free Technique

**Figure 4:**
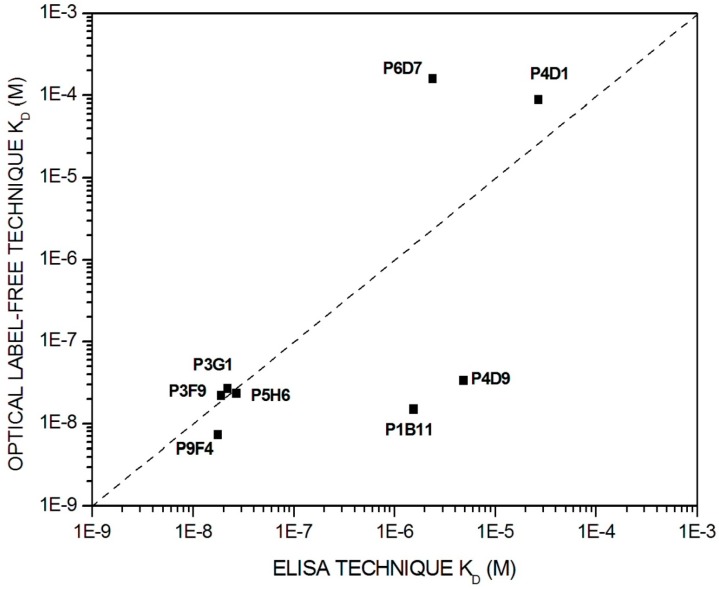
Equilibrium dissociation constant (K_D_) measurements obtained using the Elisa technique and the Optical Label-free technique.

Dissociation constant values for eight antibody-antigen systems were compared with ELISA and the Optical Label-Free technique. This analysis, shown in Figure 4, indicated that five antibodies have K_D_ values in the same order of magnitude with both techniques. However, three antibodies show dissociation constant values that differ by two orders of magnitude. 

The differences between both techniques can be justified as follows: the Enzyme-Linked Immuno Sorbent Assay (ELISA) technique is a method where affinity constants is determined in dilution and, therefore, a real immunoreaction constant is determined. However, by employing the optical interferometric technique based on BICELLs, the reaction constant is calculated in the solid-phase, leading an apparent constant in a heterogeneous biosensing assay. Moreover, the optimization of immunoassay (e.g., pH of buffers, incubation times, and temperature, among others) may have significant implications and influence the antigen-antibody interaction. For this reason, the quantitative estimation of the affinity constant with our optical interferometric technique is an essential piece of information when setting up a heterogeneous biosensing assay.

## 4. Conclusions

The affinity antigen-antibody for several biomarkers associated with dry eye disease was studied using an optical label-free interferometric technique. For this study biophotonic sensing cells (BICELLs) based on SU8 photoresist have been used.

Antibodies for three biomarkers: ANXA1, ANXA11 and PRDX5 were produced. The affinity of the antibodies was tested by the ELISA technique calculating their dissociation constant (K_D_) and therefore the affinity to their corresponding antigens.

An indirect immunoassay until antigen saturation on the sensing surface took place by an optical label-free technique was performed. Then, a recognition curve for each antibody was plotted. From this curve, an apparent dissociation constant (K_D_) was calculated and compared with the ELISA result.

In general terms, antibodies with K_D_ < 10^−7^ M have high affinity. Therefore, for the ANXA1 biomarker, two antibodies were studied by using ELISA and the optical label-free technique. As a result, both antibodies exhibited poor affinity. However, for the ANXA11 biomarker we observed a good affinity reaction: the best antibody is P3F9 for both techniques. Finally, for the PRDX5 biomarker the three antibodies also had a good affinity by both techniques.

As a main conclusion, the comparative analysis of K_D_ indicates a reasonable correlation between both techniques in some antigen-antibody pairs. However, in other pairs there are significant differences. We consider that the main different values of K_D_ between both IVD techniques are more related with the different immunoassays protocols when using ELISA in solution in comparison with the BICELLs based optical interferometric technique in heterogeneous medium. As explained above, parameters such as buffer, sample volume, incubation time, blocking steps and washing can impact the determination of the K_D_. Finally, even with the different K_D_ values observed, the proposed interferometric optical label-free technique seems to be suitable to study antigen-antibody affinity.
